# Macroclimatic and maternal effects on the evolution of reproductive traits in lizards

**DOI:** 10.1002/ece3.8885

**Published:** 2022-05-02

**Authors:** Dylan J. Padilla Perez, Michael J. Angilletta

**Affiliations:** ^1^ School of Life Sciences Arizona State University Tempe Arizona USA

**Keywords:** adaptation, energetics, fecundity, reproductive output, tradeoffs

## Abstract

Much of life‐history theory rests on fundamental assumptions about constraints on the acquisition and allocation of energy to growth and reproduction. In general, the allocation of energy to reproduction depends on maternal size, which in turn depends on environmental factors experienced throughout the life of the mother. Here, we used phylogenetic path analyses to evaluate competing hypotheses about the environmental and maternal drivers of reproductive traits in lizards. In doing so, we discovered that precipitation, rather than temperature, has shaped the evolution of the life history. Specifically, environments with greater rainfall have enabled the evolution of larger maternal size. In turn, these larger mothers produce larger clutches of larger offspring. However, annual precipitation has a negative direct effect on offspring size, despite the positive indirect effect mediated by maternal size. Possibly, the evolution of offspring size was driven by the need to conserve water in dry environments, because small organisms are particularly sensitive to water loss. Since we found that body size variation among lizards is related to a combination of climatic factors, mainly precipitation and perhaps primary production, our study challenges previous generalizations (e.g., temperature‐size rule and Bergmann's rule) and suggests alternative mechanisms underlying the evolution of body size.

## Graphical abstract

Our study indicates that precipitation is the most likely driver of the evolution of reproductive traits in lizards. We challenge previous generalizations and suggest alternative mechanisms underlying the evolution of body size.

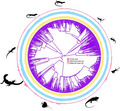

## 1 INTRODUCTION

Life‐history theory consists of models designed to explain how environmental factors shape the evolution of survival, growth, and reproduction (Roff, 2002; Stearns, 1992). Much of this theory focuses on the covariation of body size and reproductive traits, given a tradeoff between the size and number of offspring (Smith & Fretwell, 1974). The optimal covariation depends on ways in which body size affects the availability of energy, the constraints on reproduction, and the survival of offspring (De Jong & Van Noordwijk, 1992; Parker & Begon, 1986; Van Noordwijk & de Jong, 1986). Often, larger females have more resources to spare, can carry more offspring during gestation, pass larger offspring during birth, or provide better care after birth. These factors influence the constraints on or the benefit of specific reproductive tactics. For example, when the abdominal cavity and pelvic opening constrain offspring size, larger females can produce larger offspring. Conversely, a small female may be unable to produce an offspring of the optimal size when this size exceeds the physical constraint (Congdon & Gibbons, 1987; Du et al., 2005; Shine, 1992; Sinervo & Licht, 1991; Vitt & Congdon, 1978). This constraint results in a positive relationship between maternal size and offspring size (Oufiero et al., 2007), in which each female produces the largest egg possible for its body size. Natural selection can produce a similar relationship when larger females have greater surplus energy (Parker & Begon, 1986). Depending on both its foraging efficiency and the availability of resources, a larger female should use its additional energy to produce larger clutches. However, given a tradeoff between offspring size and offspring number, the investment in more offspring may be balanced by a decrease in the size of offspring. Similarly, when increasing the number of offspring increases competition among the resulting siblings, a female should use additional energy to produce larger offspring instead of more offspring. Interestingly, a mixed strategy can be optimal when intraspecific competition and offspring size both exert important influences on offspring survival; in such cases, larger females should lay larger offspring and larger clutches (Parker & Begon, 1986).

**FIGURE 1:**
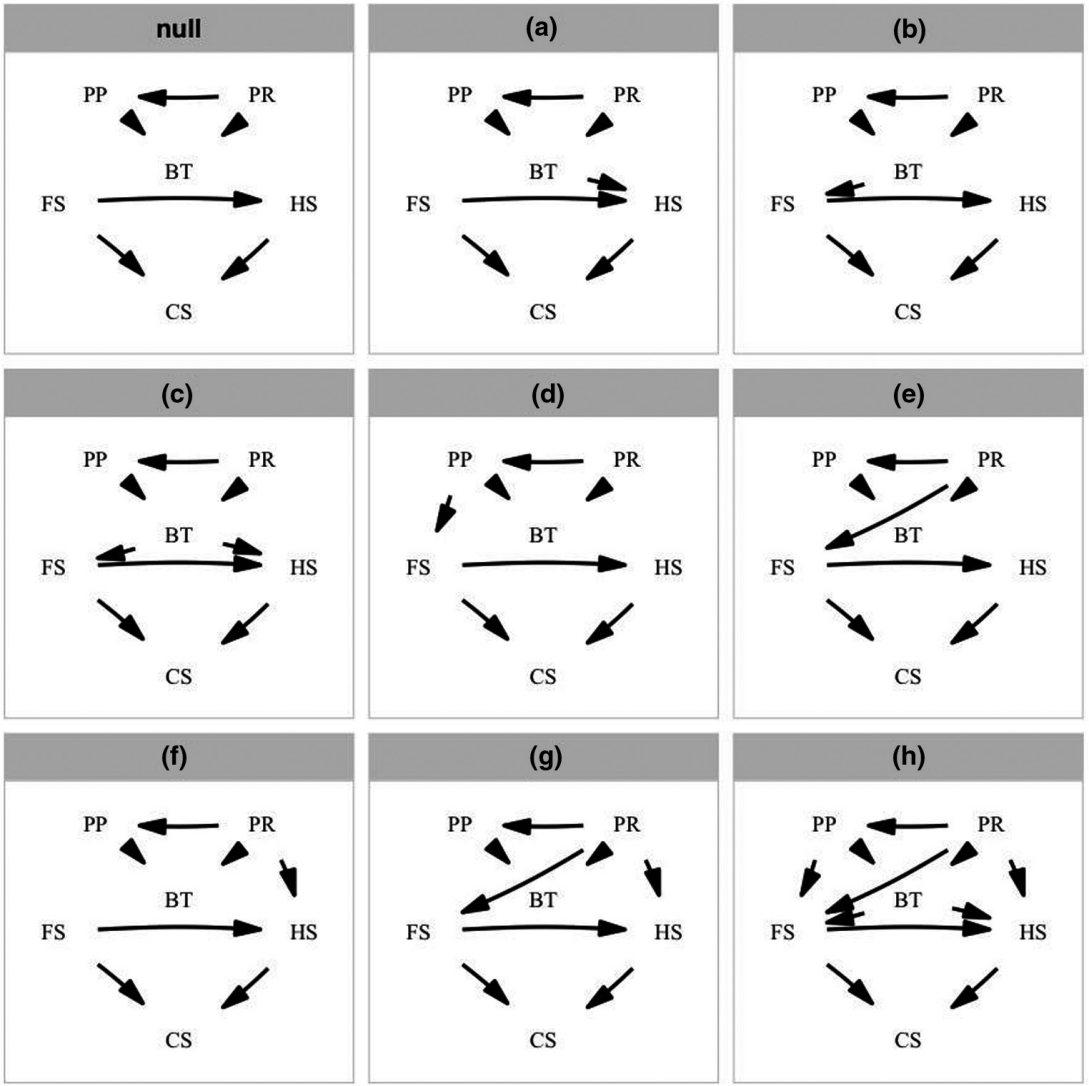
Path models depicting relationships among body temperature and life‐history traits derived from optimality models (see text for details). Abbreviation in the path diagrams are as follows: BT, body temperature; CS, clutch size; FS, maternal length; HS, hatchling/neonate length; PP, primary production; PR, annual precipitation

Because growth and body size are affected by environmental conditions, the covariation among abiotic factors should shape the evolution of life‐history traits (Figure 1). Temperature is a well‐known factor that directly and indirectly affects the evolution of the life history. The direct effect occurs when environmental conditions determine the relationship between the size and performance of offspring. Warmer environments often promote a high relative growth rate, potentially reducing the size of offspring. In such cases, a female can produce more, smaller eggs as the optimal reproductive tactic. The indirect effect occurs because temperature greatly affects the size at which an organism reproduces (Atkinson, 1994, 1995; Atkinson & Sibly, 1997). When exposed to a warmer environment, an organism may grow faster but mature younger at a smaller size, a pattern of phenotypic plasticity observed among some ectotherms (Ashton, 2002; Berrigan & Charnov, 1994; Sibly & Atkinson, 1994) and many endotherms (Ashton et al., 2000; Bergmann, 1848). If a warmer environment causes an organism to reproduce when younger and smaller, the smaller parent should make fewer or smaller offspring. In addition, variation in body size may also depend on precipitation, which is a good predictor of primary production (McNab, 2010; Yom‐Tov & Geffen, 2006). Generally, abundant precipitation and radiation increase the rate of photosynthesis (Cramer et al., 1999), which provides the energy required for growth and reproduction of organisms at higher trophic levels. If more productive areas enable organisms to consume more prey, one should expect these organisms to mature younger at smaller size; this indirect effect on maternal size should affect the size and number of offspring of the mother, as discussed earlier.

Although a covariation among environmental and maternal factors is expected to drive the evolution of the life history, most studies have focused on temperature as the main selective pressure. This bias has resulted in a wealth of conflicting results in the current literature as several comparative and species‐specific studies have found no consistent trend. For instance, an analysis of over 100 studies, Atkinson (1994) concluded that in more than 80% of the cases, decreased temperature leads to maturation later at a larger size. However, the variation among populations in natural environments paints a more complex picture. Given that environmental temperature generally increases with decreasing latitude, we should expect to observe a positive relationship between latitude and offspring size. Interestingly, a meta‐analysis by Marshall et al. (2018) revealed no general relationship between latitude and offspring size; relationships ranged from strongly negative to strongly positive. Moreover, the variation among natural populations likely reflects a combination of phenotypic plasticity and genetic divergence. A recent experiment by Kouyoumdjian et al. (2019) illustrates this point. They compared the reproductive traits of lizards from low or high elevation to that of lizards transplanted from low elevation to high elevation. Lizards from high elevation produced slightly larger eggs than did lizards from low elevation. However, lizards transplanted to high elevation produced eggs of intermediate size, although none of these differences were significant. Thus, the evolution of egg size might be driven partly by genetic factors, as well as environmental conditions during early development. Moreover, body size has diverse effects on reproductive traits, when analyzed independently of temperature. Larger females increase annual biomass production (Meiri et al., 2012), produce larger hatchlings (Le Gouvello et al., 2020), or more hatchlings (Warner & Shine, 2008). If these diverse patterns reflect diverse processes among taxa, a model focused on a single mechanism would be unable to explain variation in reproductive traits. Therefore, a unified approach to examine the combined effects of different environmental and maternal factors on the broad‐scale variation in reproductive traits might provide the best insight.

Phylogenetic path analysis is an effective way to test hypotheses about multiple causes of evolutionary patterns (Gonzalez‐Voyer & Hardenberg, 2014; Hardenberg & Gonzalez‐Voyer, 2013). Previously, Angilletta et al. (2006) combined path modeling, phylogenetic analysis, and information theory to infer the most likely relationships among environmental temperature, body size, and reproductive traits for 19 populations of sceloporine lizards. They found that temperature likely affects the number and size of offspring indirectly, via its effect on maternal body size. Since then, other researchers compiled published data for body sizes and reproductive traits of several species of lizards (Meiri, 2008, 2018; Meiri et al., 2012, 2015; Warne & Charnov, 2008). Here, we use these data to evaluate competing hypotheses about the evolution of reproductive traits through phylogenetic path analyses (Figure 1). In doing so, we found that annual precipitation is the major driver of the life history. By using phylogenetic path analysis, we suggest a more general mechanism underlying the evolution of life‐history traits of lizards.

## 2 MATERIALS AND METHODS

### 2.1 Data source

We used published estimates of life histories for 486 species of lizards, belonging to 34 families (see supporting code). We used the mean snout‐vent length for hatchlings or neonates (mm), and adult females, as a measure of body size among species. Similarly, we averaged the lowest and highest reported means of clutch or litter sizes for each species (see Meiri (2018) for details). We used the length of hatchlings as an estimate of offspring size, rather than the mass of eggs, because the latter might reflect variation in water content rather than energy content in species of lizards that lay poorly calcified eggs (Deeming et al., 2004; Meiri et al., 2015). Moreover, hatchling size can be estimated for viviparous species as well as oviparous species. Importantly, when female mass and hatchling mass are included in the analyses, the results of the study remain consistent (see supporting code).

Because the range of nearly all reptiles have been recently mapped (Roll et al., 2017), we could obtain data on annual air temperature and annual precipitation across the range of each species. These climatic variables derived from the monthly values of temperature and rainfall for global land areas at a resolution of 30 s (Fick & Hijmans, 2017). Similarly, we obtained data on log‐transformed values of net primary production—the net amount of solar energy converted to plant organic matter through photosynthesis—measured in units of elemental carbon. This measure represents the primary source of trophic energy for the world's ecosystems (Imhoff et al., 2004). We used polygonal range maps—representing species extent of occurrence—to run a zonal statistical algorithm in the software QGIS version 3.22.2‐Białowieża; this algorithm enabled us to extract the means of climatic data across pixels of a map for each species. In addition to extracting climatic data, we also gathered published records of body temperature measured in the field for 320 species. These data consist of an average of the minimal and maximal temperatures reported in the literature (Meiri, 2018).

### 2.2 Statistical analyses

We used Phylogenetic Path Analysis (van der Bijl, 2018) to test our hypotheses on the relationships among macroclimatic factors, maternal factors, and reproductive traits of lizards. In comparative biology, normal regression models cannot be used for path analysis as species are part of a hierarchically structured phylogeny, and thus cannot be regarded for statistical purposes as if drawn independently from the same distribution (Felsenstein, 1985; Harvey et al., 1991). Phylogenetic path analysis corrects for similarity by descent to reveal relationship that might be attributed to evolutionary processes. Following Hardenberg and Gonzalez‐Voyer (2013), we used Phylogenetic Generalized Least‐squares (PGLS) to perform confirmatory path analysis based on the directed separation method (“d‐separation”; Shipley, 2013). This phylogenetic path analyses were informed by a recent time‐calibrated phylogeny for squamate taxa (Zheng & Wiens, 2016).

We tested our hypotheses (see Figure 1) by performing phylogenetic path analysis of data of body temperature, and environmental temperature. Examining the explanatory power of competing models involving different relationships among variables enabled us to identify the most plausible hypothesis given the available data, and thus infer the relative importance of mechanisms described by optimality models. These analyses were performed with the *phylopath* library of the Statistical Software R version 4.0.5 (R Core Team, 2013). We calculated each model's goodness of fit with the Fisher's C statistic (Shipley, 2000), obtained as follows:

$$C=‐2\sum_{i=1}^{k}\ln{(p}_{i})$$

where *k* is the number of conditional independencies in the minimal set and *p_i_* is the null probability associated with each of the predicted independence claims tested. A conditional independency specifies the list of pairs of variables that are statistically independent conditioning on a set of other variables in the causal model. For example, a conditional independency that supports one of our hypotheses relates body temperature (BT), maternal size (FS), and clutch size (CS; see Figure 1b). In this example, clutch size is d‐separated from body temperature, which means that there is not an arrow linking these variables (or “vertices”) in the directed acyclic graphic. Maternal size is considered the causal “parent” of both body temperature and clutch size because it is the variable directly linked with both vertices (BT and CS). It is possible now to translate these d‐separation statements to statistical linear models in which we test the independence of clutch size and temperature, conditioning on their parent, maternal size. The C statistic is a maximum likelihood estimate that follows a *x* ^2^ distribution with degrees of freedom *df* = 2k. Therefore, it provides a convenient statistic for testing the goodness of fit of the whole path model (Shipley, 2013). The path model does not provide a good fit to the data if the *p* value of the *C* statistic is below the alpha value (.05). We compared models using a modified version of the Akaike Information Criterion (AIC) known as the *C* statistic Information Criterion (CIC), proposed by Hardenberg and Gonzalez‐Voyer (2013). Here, we calculated the CIC_c_, which is the equivalent of CIC with a correction for small sample sizes, as follows:

$${\mathrm{CIC}}_{c}=C+2q\frac{n}{\left(n‐1‐q\right)}$$

where *C* is the maximum likelihood of the particular model, *q* is the number of parameters estimated in the path model, and *n* is the sample size (number of species). The most likely model has the lowest CIC*_c_* value (Burnham & Anderson, 2002).

Our data analyses were entirely based on full model averaging, by which one calculates a likelihood‐weighted average of parameters among all models ranked. Model averaging causes the path coefficients that do not occur in all models to shrink toward zero (van der Bijl, 2018). We reported the conditional independencies that supported the averaged model. We also reported the *p*‐values of each independence statements, and an estimate of the Pagel's *λ* to account for phylogenetic nonindependence of each statement (see supporting code). The parameter *λ* can vary between 0 and 1; a value of 0 for a conditional independency indicates that the relationship between the life histories involved is not constrained by the phylogeny, whereas a value of 1 indicates the converse.

## 3 RESULTS

**FIGURE 2:**
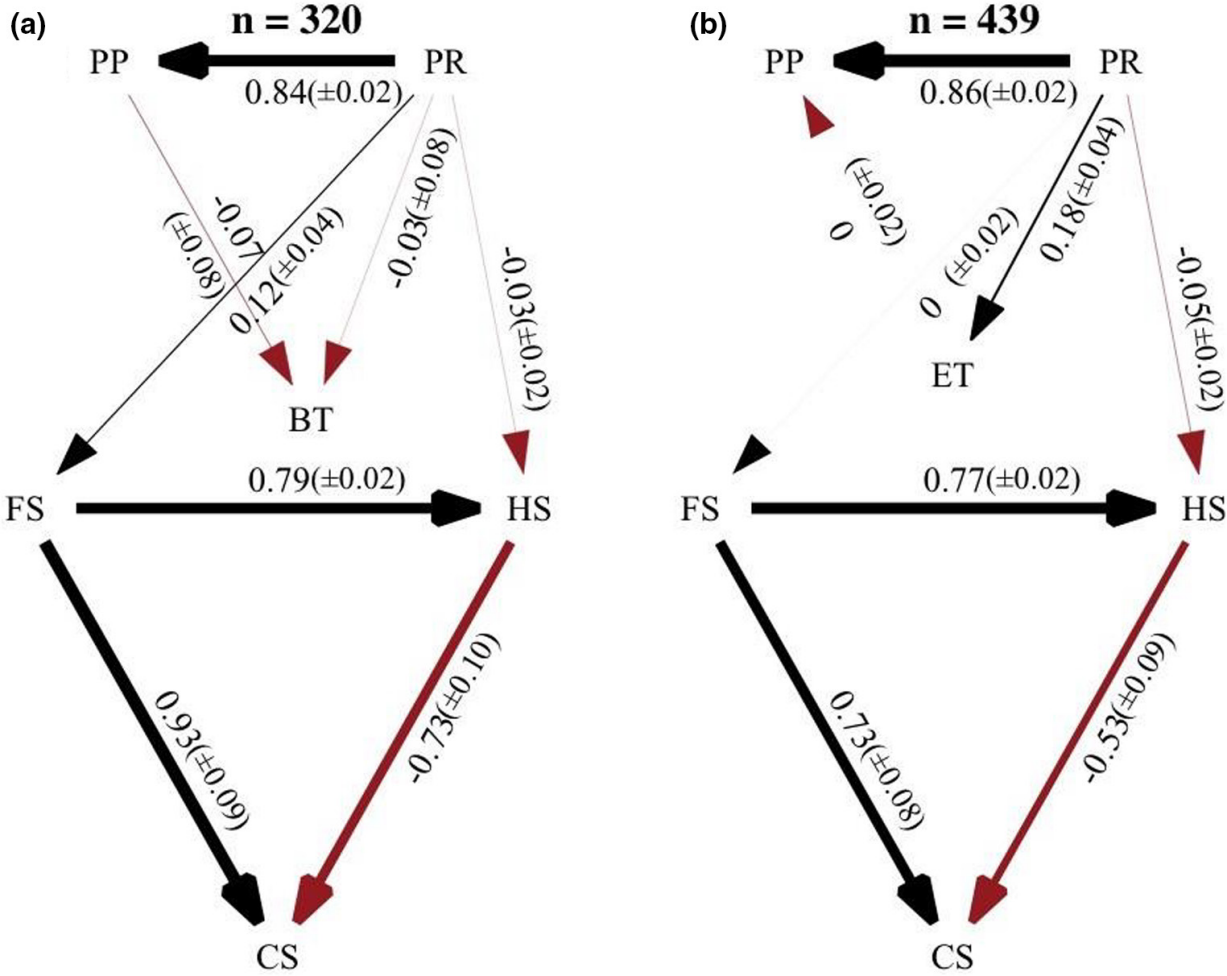
Best‐fit models of the evolution of reproductive traits considering body temperature (a) and environmental temperature (b) as independent variables. Thicker arrows indicate stronger effects. Values in parentheses denote the standard errors of the path coefficients

Phylogenetic path analyses revealed that annual precipitation directly and indirectly affected the evolution of the reproductive traits in lizards (Figure 2). The direct effect stems from its negative relationship with hatchling size, while the indirect effect is mediated by its effect on maternal size. These analyses also indicate that body temperature had little or no effect on the evolution of reproductive tactics, either directly or indirectly. We drew this conclusion from the coefficients derived from full model averaging, which indicated negligible effects of body temperature on maternal size and hatchling size (Figure 2a). Importantly, we found a similar pattern when using the mean environmental temperatures across the species range as an independent variable in our analysis (Figure 2b).

**FIGURE 3:**
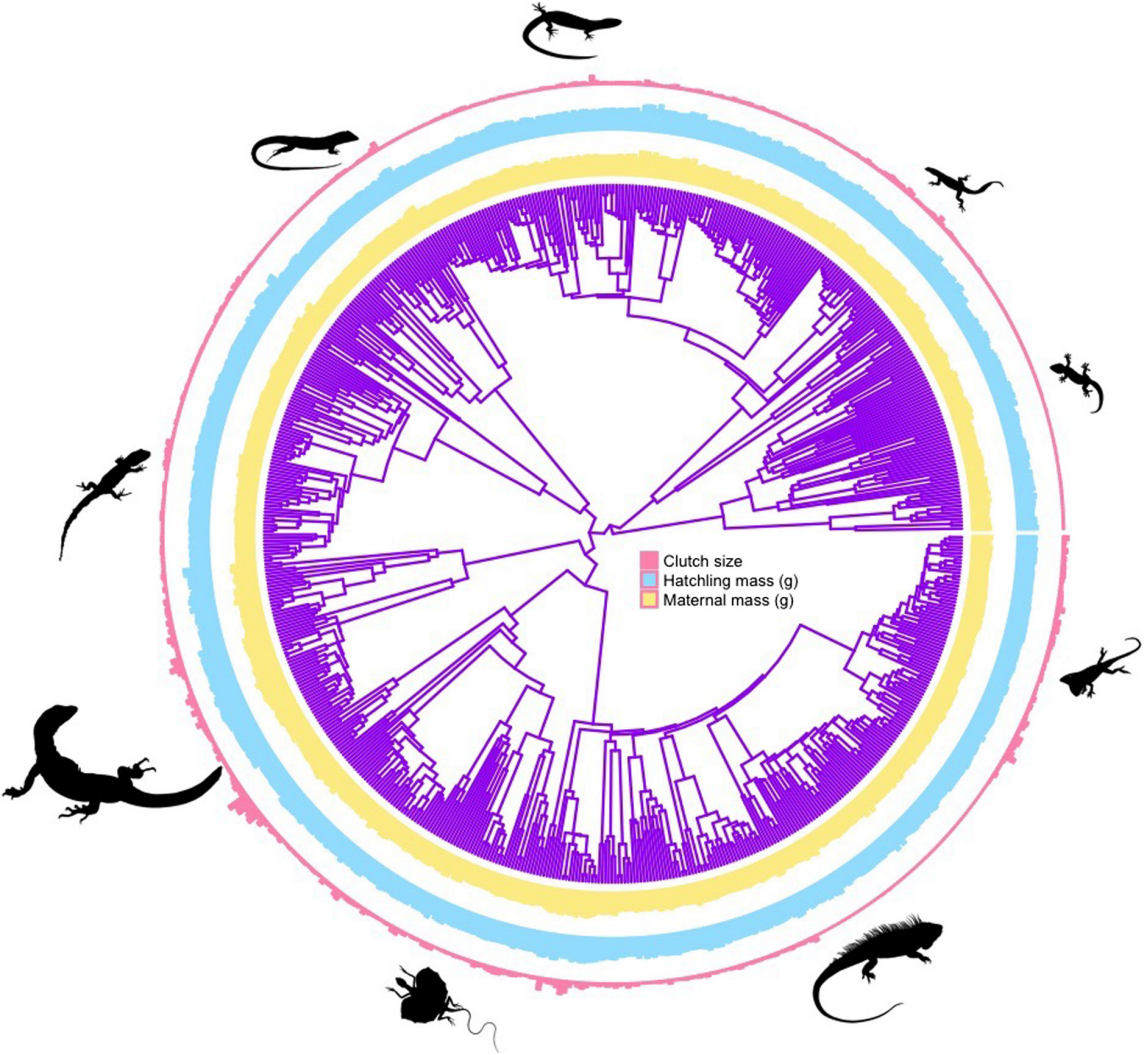
Evolution of the reproductive output among 669 species of lizards. The dataset used to perform path analyses was reduced to 486 species because we could not extract data of climatic variables for all species present in the phylogeny

Our analyses also revealed that maternal size greatly determined the evolution of hatchling size and clutch size. Specifically, larger females simultaneously produce larger offspring and more offspring (Figure 2). By accounting for maternal size in our phylogenetic path analysis, we observed the expected negative relationship between hatchling size and clutch size. Likely, larger females produce larger clutches of larger offspring by having more surplus energy to allocate to reproduction (Figure 3).

## 4 DISCUSSION

**FIGURE 4:**
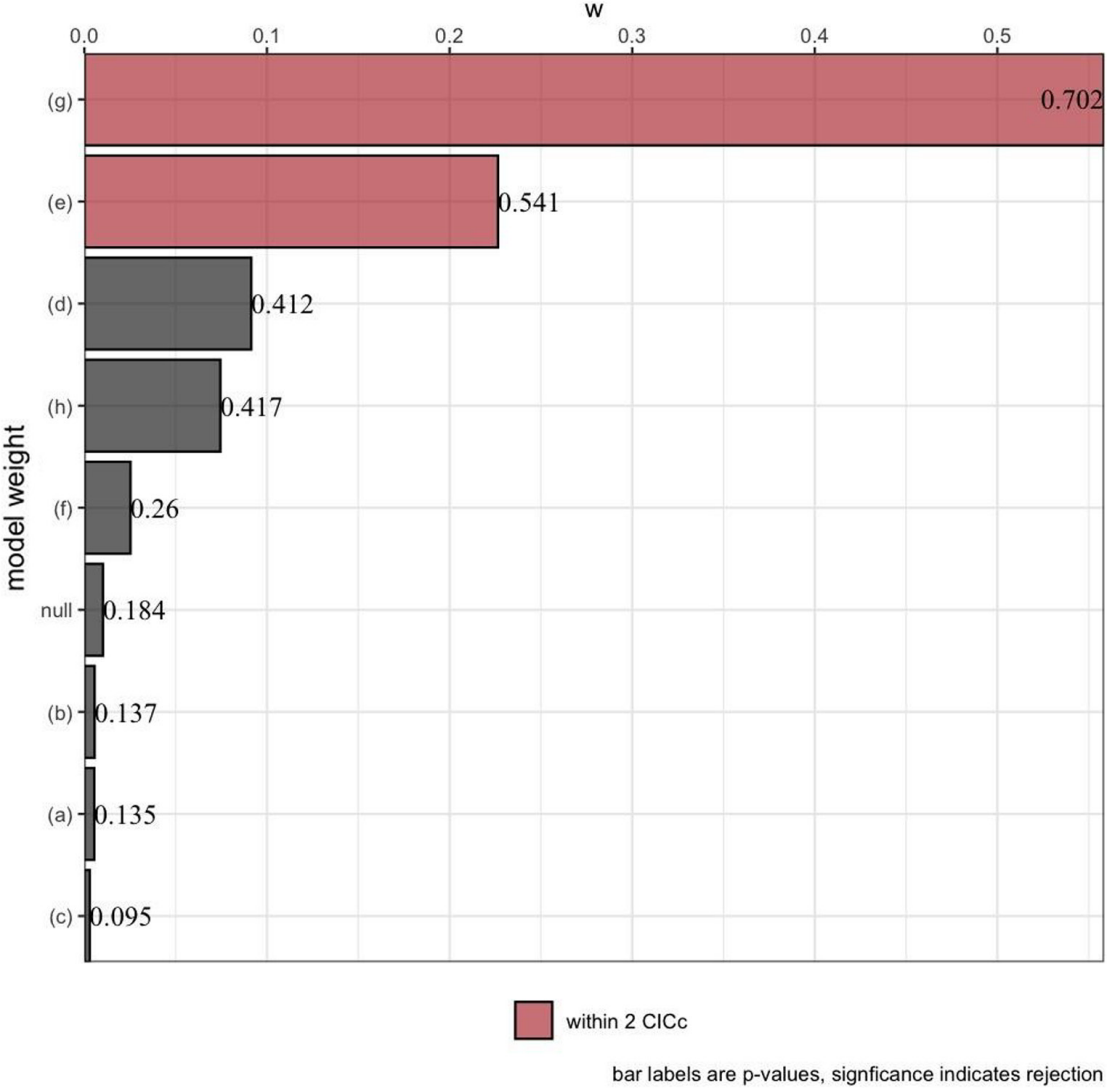
Relative importance of the causal models describing the evolution of reproductive traits in lizards

A model describing a direct and an indirect effect of precipitation on the evolution of reproductive traits was strongly supported (see Figure 1g). On one hand, we found a negative relationship between precipitation and hatchling size. On the other hand, we found a positive effect of precipitation on maternal size. Both relationships might reflect selective pressures on body size associated with food availability. Greater precipitation often results in greater primary production, which translates to greater food abundance for lizards (McNab, 2010; Yom‐Tov & Geffen, 2006). In a more productive environment, a juvenile lizard could mature younger at a smaller size. Additionally, given the tradeoff between the size and number of offspring, a mature lizard should produce more, smaller offspring in an environment that favors growth. Although the averaged model indicates no strong direct effect of primary production on maternal size (Figure 2a), our analysis revealed that a model including this path should also be considered important based on the ∆CIC*_c_* (Figure 4). Alternatively, we consider other mechanisms by which precipitation can directly influence body size. The water‐conservation hypothesis provides one such mechanism. This hypothesis predicts stronger selection for large size in dry environments, given that the size specific rate of water loss decreases with increasing size (Gouveia & Correia, 2016; Nevo, 1973; Olalla‐Tárraga et al., 2009; Pincheira‐Donoso et al., 2008).

The absence of a thermal effect on the evolution of reproductive tactics might partially be explained by thermoregulation. Many species of lizards use behavior and physiology to buffer variation in environmental temperature (Huey et al., 2003). This buffering ability reduces selective pressures on thermal physiology, slowing evolutionary divergence over space and time (Bogert, 1949; Buckley et al., 2015; Huey et al., 2003). The conservation of thermal tolerance within species has been widely recognized (Buckley et al., 2015; Van Damme et al., 1990), but this phenomenon is also evident among some species of lizards (Cruz et al., 2009; Sunday et al., 2011). Body temperature constrains life histories because it exerts an important influence on the acquisition of energy required for growth and reproduction. Normally, the detection, capture, ingestion, digestion, and assimilation of food are processes that determine the acquisition of energy. These processes perform well over an optimal range of body temperatures and perform poorly at temperatures outside this optimum (Huey, 1982). Whether a lizard's optimal temperature for performance remains similar in space and time depends on the effectiveness of thermoregulation. A thermoregulating lizard might bask in open areas more often at high altitudes or latitudes, but it might use shaded areas more often at low altitudes or latitudes (Adolph, 1990; Rand, 1964). Consequently, the optimal body temperatures of species from different altitudes or latitudes might not change drastically (Huey et al., 2003). If this behavior enabled some lizards to maintain their body temperature close to the thermal optimum regardless of the climate, thermoregulation would have slowed the divergence of life‐history traits among species. Because nocturnal lizards have fewer opportunities to behaviorally thermoregulate than diurnal lizards do, we expect temperature to have a greater effect on the reproductive tactics of these lizards.

Additionally, local adaptation of thermal physiology among species might reduce the impact of environmental temperature on the life history of lizards. If the preferred body temperatures of species have diverged to track the environmental temperatures of their habitats, natural selection would favor a subsequent shift in the thermal optima for performance (Huey & Bennett, 1987). This coadaptation of thermoregulatory behavior and thermal physiology would enable each species to perform best at the body temperature commonly experienced in its environment. In lizards, Bauwens et al. (1995) found a significant correlation between the preferred temperature and the thermal optima for sprint speed among 13 species of lacertids. Similarly, Huey and Kingsolver (1993) found that thermal optima for sprinting correlated strongly with field body temperature among 19 species of iguanid lizards. Thus, the coadaptation of thermoregulatory behavior and thermal physiology seems a likely factor contributing to the patterns of interspecific variation that we observed in general among lizards.

The evidence that females produce larger clutches of larger offspring accords with certain optimality models in which natural selection maximizes the reproductive success of the parent (Roff, 2002). This positive relationship between maternal size and clutch size, or fecundity, mirrors those observed within most species (Parker, 1970; Ridley & Thompson, 1979; Roff, 2002). Presumably, larger females lay larger clutches because they have more surplus energy (Parker & Begon, 1986). However, we also found that larger mothers produce larger offspring. For this strategy to be optimal, the size of the mother must affect the relationship between the size and fitness of offspring (Smith & Fretwell, 1974). Several hypotheses have been proposed to explain why larger mothers should produce larger offspring (Marshall et al., 2010; Sakai & Harada, 2001). Following Taylor and Williams (1984), Sargent et al. (1987) showed that mothers should produce larger offspring when offspring size promotes growth and reduces mortality. They interpreted this result to mean that larger females should produce larger eggs whenever they provide better parental care, which increases growth or reduces mortality. In support of this model, seed beetles produced larger offspring when they were able to lay their eggs on better‐defended seeds (Fox et al., 1997). But do larger species of lizards provide better parental care for their offspring? Possibly, larger mothers deter more predators and provide more food for their offspring. Consistent with this idea, a large species of skink, *Eutropis longicaudata*, better deterred snakes from eating their eggs than did a small species, *Sphenomorphus incognitus* (Huang, 2006; Tseng & Huang, 2012). Still, more evidence is needed to determine whether parental care, other than energy provisioning, plays an important role in the growth and survival of juveniles, particularly for oviparous lizards that do not interact with their offspring after laying eggs in a nest. Alternatively, larger mothers might produce larger offspring because of competition among siblings; this strategy simultaneously reduces the intensity of competition (a smaller clutch) and increases the competitiveness of offspring (larger offspring). Both of these models could explain the positive relationship between maternal size and offspring size that we observed among species of lizards.

These models of optimal reproductive allocation depend on a fundamental trade‐off between the number and size of offspring (Roff, 2002; Smith & Fretwell, 1974; Warne & Charnov, 2008). We found strong evidence for this tradeoff among species. One's ability to detect a tradeoff between the size and number of offspring depends on the variation in energy supply among mothers (Van Noordwijk & de Jong, 1986). Controlling for body size should reveal the expected tradeoff between the size and number of offspring. However, some families of lizards provide evidence of exception for this tradeoff, such as Chamaeleonidae and Varanidae (Figure 3). For example, Diaz‐Paniagua et al. (2002) showed that egg characteristics (length, width, and mass) remained constant despite variation observed in clutch size and maternal body length and mass of chameleons. This finding supports the idea of an optimal egg size in chameleons (Smith & Fretwell, 1974). According to the optimal egg size theory, clutch size, rather than egg and offspring size, varies with fluctuating resource availability. If offspring size does not depend on clutch size or maternal size, such that large females produce the same size of eggs as small females do, the expected offspring size and offspring number tradeoff might be muted. These exceptions show the importance of analyses such as ours, which can help determine whether body size drives resource availability through comparative evidence.

Our study indicates that precipitation is the most likely driver of the evolution of reproductive traits in lizards. Although biologists have intensely debated the mechanisms by which temperature affects body size (Angilletta, 2009; Atkinson & Sibly, 1997; Partridge & French, 1996), we found no consistent relationship between temperature and body size among species of lizards. Interestingly, we now know that lizards with larger amounts of reproductive resources produce both larger hatchlings and larger clutches. Although we do not know the selective pressures responsible for this pattern, theoretical models tell us that parental care or intraspecific competition could play a role (Parker & Begon, 1986). In lizards, we lack experimental evidence that larger females provide better parental care. We also do not know the relationship between offspring size and competitive ability for most species. Therefore, empirical evidence of this nature would clarify whether these mechanisms play any role in the evolution of life histories in lizards. For longer lived species such as lizards, experimental studies of life‐history evolution have lagged behind comparative studies. Still, phylogenetic path analysis has enabled us to disentangle some evolutionary relationships among life histories. This analysis sharpens our focus on mechanisms underlying major evolutionary patterns, stimulating the development of a more general life‐history theory.

## CONFLICT OF INTEREST

We declare we have no conflict of interests.

## AUTHOR CONTRIBUTIONS

**Dylan J. Padilla Perez:** Conceptualization (equal); Formal analysis (equal); Funding acquisition (equal); Writing – original draft (equal); Writing – review & editing (equal). **Michael Angilletta:** Supervision (equal); Writing – original draft (equal); Writing – review & editing (equal).

## Data Availability

For access to the data associated with our study, use the following link: https://doi.org/10.5061/dryad.9p8cz8wjn.
